# Cardiovascular Risk Associated With TNF Alpha Inhibitor Use in Patients With Rheumatoid Arthritis

**DOI:** 10.7759/cureus.17938

**Published:** 2021-09-13

**Authors:** Aaiz Hussain, Targol Tarahomi, Lavi Singh, Murali Bollampally, Milad Heydari-Kamjani, Marc M Kesselman

**Affiliations:** 1 Dr. Kiran C. Patel College of Allopathic Medicine, Nova Southeastern University, Davie, USA; 2 Dr. Kiran C. Patel College of Osteopathic Medicine, Nova Southeastern University, Davie, USA; 3 College of Liberal Arts and Science, Wayne State University, Detroit, USA; 4 College of Osteopathic Medicine, Michigan State University, East Lansing, USA; 5 Department of Internal Medicine, Cleveland Clinic Florida, Weston, USA; 6 Division of Rheumatology, Dr. Kiran C. Patel College of Osteopathic Medicine, Nova Southeastern University, Davie, USA

**Keywords:** rheumatoid arthritis, heart failure, tnf alpha inhibitor, tnf alpha, congestive heart failure

## Abstract

Rheumatoid arthritis (RA) is an autoimmune disease characterized by inflammation and pannus formation, with subsequent joint and cartilage degradation. Treatment commonly targets inflammatory cytokines, including tumor necrosis factor (TNF) alpha, which is a potent inflammatory cytokine required for cell signaling, regulation, and apoptosis, as well as for other cellular functions including immune response. TNF alpha inhibitors have demonstrated benefits in improving RA patient outcomes in terms of immune function and symptomatology. While TNF alpha inhibitors are generally beneficial, some studies have demonstrated that TNF alpha inhibitors may increase the risk of adverse cardiovascular events. While this continues to be debated, our study investigates the role of Tumor Necrosis Factor Receptor 1 (TNFR1) and Tumor Necrosis Factor Receptor 2 (TNFR2) in cardiac tissue. TNFR1 is an apoptotic receptor and its inhibition by TNF alpha inhibitors is subsequently cardioprotective. However, TNF alpha inhibitors may be inhibiting TNFR2 receptors even more so than TNFR1 receptors. TNFR2 is primarily a cardioprotective receptor and its greater inhibition results in the cardiovascular morbidity associated with TNF alpha inhibitors.

## Introduction and background

Globally, rheumatoid arthritis (RA) is a chronic, inflammatory autoimmune disease that affects one percent of the population [1]. In the United States, the prevalence of RA is between 1.28 and 1.36 million adults [2]. RA is a symmetric and progressive polyarthropathy affecting the joints, which can present with extra-articular manifestations including accelerated cardiovascular disease [3]. Various risk factors for the development of RA include age, sex, and genetic alterations (i.e. HLA DR4, IL23R, TRAF1, CTLA4, IRF5, STAT4, CCR6, PADI4 subtype, PTPN22) [4]. In a recent study, the mortality rate of RA cases was 26.90 per 1000 person-years (95% CI 25.87- 27.97) and 18.92 (18.48-19.36) per 1000 person-years for controls [5]. RA can further be complicated by the high prevalence of comorbid cardiovascular disease (35%, according to a systematic review) in the United States [6]. It has been estimated that RA patients have a 1.5-2 fold increased risk of developing cardiovascular events, and up to 50 percent, when compared to the general population [7]. The pathogenesis behind this increased susceptibility remains unclear, but it is thought to be a complex interplay of pre-existing traditional cardiovascular risk factors and a state of chronic inflammation [8]. TNF alpha is one of many pro-inflammatory cytokines that has been suspected to play a pivotal role in the pathogenesis and development of cardiovascular disease and heart failure (HF) in RA patients [8]. TNF alpha is not constitutively expressed in the myocardial tissue [9]. However, upon induction of stressful events such as myocardial infarction, its level of expression is increased, which has allowed research studies to identify its role as a negative inotrope affecting cardiac tissue remodeling [10]. This is an important aspect of treating patients with RA, as these patients have a higher rate of cardiovascular events compared to the general population. Patients with RA are systemically predisposed to cardiovascular risk factors including dyslipidemia, vascular inflammation, and high levels of TNF alpha. As such, this review will provide insight into the effect of TNF alpha inhibitor therapy on cardiovascular health in patients with RA, outlining current standards of treatment and cardiovascular event risk. Furthermore, the pathophysiology and the subsequent downstream effect of TNF alpha on cardiac tissue through Tumor Necrosis Factor Receptor 1 (TNFR1) and Tumor Necrosis Factor Receptor 2 (TNFR2) will be investigated [11,12].

## Review

Heart failure and rheumatoid arthritis

Congestive heart failure (CHF) is a disease characterized by cardiac pump dysfunction [13]. A variety of etiologies can lead to CHF including cardiomyopathies, infection, neoplasm, and drugs [13]. This dysfunction leads to congestion and low perfusion, which activates a cascade of other physiological issues [13]. As a compensatory response, the kidneys detect low perfusion through renal arterioles, and the renin-angiotensin-aldosterone system (RAAS) is consequently activated, which increases volume retention [13]. This further exacerbates the work the heart needs to perform to perfuse blood throughout the body [13]. Hypertension is the primary risk factor contributing to the development of CHF, with diabetes, age, and sex contributing as well [14]. It is estimated that over six million people in the United States are affected by CHF and many succumb to its pathological effects [15].

Diastolic dysfunction is defined as a preserved ejection fraction with decreased compliance as well as impaired relaxation, most often due to hypertrophy [16]. This type of HF generally results from hypertension, as the heart works harder to pump blood against the increased vascular resistance [16]. The consequent concentric thickening of the heart results in reduced filling during diastole, reducing overall cardiac output [16]. Systolic dysfunction, conversely, is defined as a reduced ejection fraction of less than 40 percent in the setting of decreased contractility, for example, due to dilated cardiomyopathy [17]. This most often occurs due to dilation of the left ventricle due to eccentric increase in myocytes [17]. The dilation results in decreased cardiac output due to an overall decrease in pressure that the ventricle can apply [17]. In a cross-sectional study by Schau et al., the researchers discussed the prevalence of diastolic HF in patients with RA, and identified an association of active inflammation in patients with RA and CHF [18]. When looking at the inflammatory markers in the patients, they saw an erythrocyte sedimentation rate (ESR) of >16 mm/hour and a C-reactive protein (CRP) of >10 mg/L (odds ratio [OR] 5.4, 95% CI 2.1-16, and 2.6, 95% CI 0.8-8.0) [18].

Rheumatoid arthritis treatment and cardiovascular risk

First-line standard treatment for patients with RA involves the use of disease-modifying anti-rheumatic drugs (DMARDs), such as methotrexate, a dihydrofolate reductase inhibitor, to decrease inflammatory response and slow disease progression [19]. However, DMARDs like methotrexate and leflunomide are not always found to be effective for RA [20]. In a comparative study done between leflunomide and methotrexate, it was found that the failure rates of leflunomide and methotrexate were 55.5 percent and 57.3 percent, respectively, with median failure time of 15 and 14 months [20]. The overall rate of DMARD discontinuation post-trial was 68.7 percent of the failure rate [20]. For patients who fail or do not adequately respond to first-line treatment, TNF alpha inhibitors are commonly prescribed [21]. TNF alpha inhibitors have demonstrated benefits in improving patient outcomes in terms of immune function and symptomatology [21].

While TNF alpha inhibitors result in inflammatory protection in most organs by decreasing levels of chronic inflammation and inhibiting the apoptotic pathway, studies have found that TNF alpha inhibitors may worsen HF in patients with systolic or diastolic dysfunction, especially at higher doses [22]. In particular, clinical trials including Research into Etanercept Cytokine Antagonism in Ventricular Dysfunction (RECOVER) and Randomized Etanercept North American Strategy to Study Antagonism of Cytokines (RENAISSANCE) tested the impact of TNF alpha inhibitors on the clinical status of HF patients [23]. In the RECOVER trial, 373 patients with HF received placebo, 375 patients received subcutaneous etanercept in doses of 25 mg every week, and 375 patients were given 25 mg twice per week for a total of 24 weeks [23]. In the RENAISSANCE trial, 309 patients received placebo, 308 received etanercept 25 mg twice per week, and 308 patients received etanercept 25 mg 3 times per week [23]. RECOVER and RENAISSANCE demonstrated that etanercept had no impact on the clinical status in patients with class II, III, or IV HF compared with control subjects [23]. Still, other studies have demonstrated that TNF alpha inhibitors may increase the risk of adverse cardiovascular events [24]. The Anti-TNF Alpha Therapy Against Congestive Heart Failure (ATTACH) trial demonstrated higher rates of HF-related hospitalization or death in the patients with New York Heart Association class III or IV HF receiving infliximab, a TNF alpha inhibitor [24]. In this 28-week trial, 50 patients with chronic HF and less than 35 percent ejection fraction were given 5 mg/kg of infliximab, 51 were given a 10 mg/kg dose, and 49 were given a placebo at weeks 0, 2, and 6 [24]. The patients that were given the 10 mg/kg dose of infliximab had an increased rate of cardiac-related hospitalization through the 28-week interval [24]. Meanwhile, for patients receiving 5 mg/kg of infliximab, a modest increase in ejection fraction was found [24]. Thus, infliximab of more than 5 mg/kg is contraindicated in patients with moderate-to-severe CHF, and if infliximab must be used, it should not exceed 5 mg/kg, and patients must be followed closely [25]. Based on the available data, the American College of Rheumatology currently recommends that TNF alpha inhibitors should be cautiously used in patients with CHF, especially in those with a reduced ejection fraction of approximately 40 percent from a healthy ejection fraction of between 50 and 70 percent [26].

TNF alpha receptors and rheumatoid arthritis treatment

**Figure 1:**
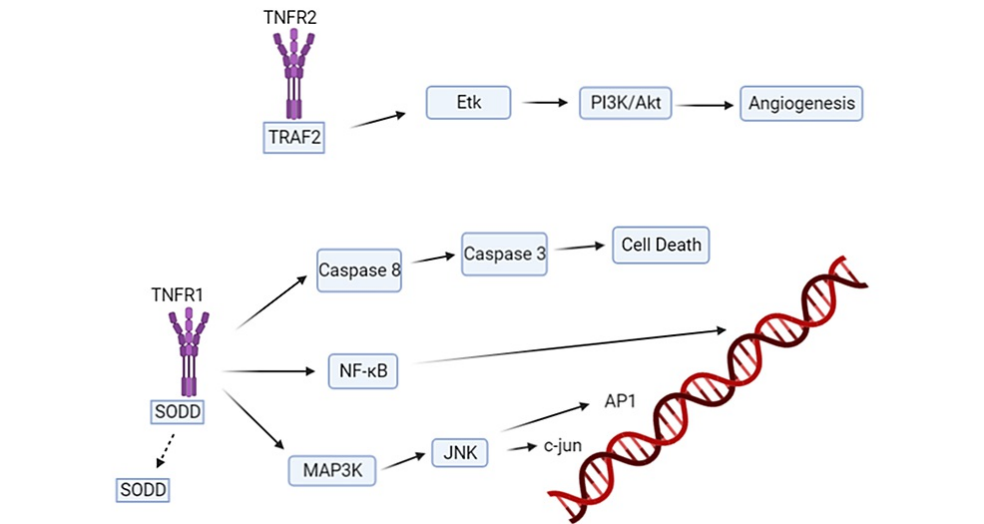
Tumor Necrosis Factor alpha-initiated signal transduction. The Tumor Necrosis Factor Receptor 1 is involved in the apoptotic pathway whereas Tumor Necrosis Factor Receptor 2 is anti-apoptotic through the Etk protein. TNF: tumor necrosis factor; SODD: silencer of death domains; TNFR1: tumor necrosis factor receptor 1; TNFR2: tumor necrosis factor receptor 2; NF-kB: nuclear factor kappa B; MAP3K: mitogen-activated protein kinase kinase kinase; JNK: c-Jun N-terminal kinase

As previously noted, RA is managed using anti-inflammatory agents such as nonsteroidal anti-inflammatory drugs (NSAIDs), DMARDs, glucocorticoids, and TNF alpha inhibitors [19]. DMARDs followed by TNF alpha inhibitors are the standard course of treatment for patients with RA [19]. TNF alpha inhibitors have been very effective in controlling RA disease activity and progression; however, the risk of cardiovascular morbidity and mortality has been debated, especially as patients with RA are already at an elevated risk for the development of cardiovascular complications [10]. Studies have shown that higher doses of TNF alpha inhibitors may contribute to the worsening of HF and reduction of patient’s lifespan [27]. TNF alpha inhibitors may play a detrimental role in cardiac myocytes and certain TNF alpha receptors have been implicated [28]. Tumor Necrosis Factor Receptor 1 (TNFR1), involved in apoptosis, and Tumor Necrosis Factor Receptor 2 (TNFR2), which has cardioprotective properties, have been implicated in Ca2+ signaling, reactive oxidative species production, and cell survival in cardiac myocytes [28]. TNF alpha binding to the TNFR1 receptor leads to disassociation of the inhibitory protein silencer of death domains (SODD) [28]. The disassociation results in activation of protein kinases and caspases (Fig.1). These effects disrupt Ca2+ homeostasis leading to disruption of the excitation-contraction mechanism within cardiac myocytes [28]. Furthermore, studies have shown desensitization of the beta-receptor on cardiac myocytes and further damage from reactive oxidative species (ROS) production [28]. Studies show that cardiac myocytes exposed to TNF alpha in vitro undergo apoptosis [29]. However, in TNFR1-deficient mouse hearts treated with TNF alpha, negligible apoptosis is observed due to sole TNFR2 upregulation and activation [28]. TNFR2 uses Etk, which is an endothelial and epithelial tyrosine kinase that promotes cell adhesion, proliferation, angiogenesis, and survival [28]. With TNFR1 having strong apoptotic and negative inotropic effects, it has been hypothesized that TNF alpha inhibitors should have therapeutic effects in patients with cardiovascular disease [30]. Yet, TNF alpha inhibitors, when given to patients with symptomatic HF, have not demonstrated significant benefits in randomized and placebo-controlled clinical trials [30]. One explanation is the inhibition of TNFR2, which has been implicated in cell survival.

In the clinical trial ATTACH, patients given the higher dose of 10 mg/kg of infliximab experienced a greater rate of cardiac-related hospitalization [24]. This may be due to a greater inhibition of TNFR2 caused by the higher dose of a TNF alpha inhibitor, which may have exacerbated cardiac failure. Conversely, patients given the lower dose of 5 mg/kg of infliximab experienced mild clinical improvement of their ejection fractions [24]. The contrast in hospitalization rates in this trial can be explained by the difference in doses administered, causing different degrees of TNFR2 inhibition. Thus, selective TNFR1 inhibitors or TNFR2 agonists may be potential treatment modalities that may allow for reduction in the detrimental effects of chronic inflammation due to RA or other immune diseases on causing damage to cardiac myocytes [30]. Two other explanations for the aforementioned phenomenon of TNF alpha inhibitors not reducing inflammatory effects on cardiac myocytes are 1) the polymorphism of the TNF alpha protein and 2) the TNF alpha inhibitors interaction with the pharmacodynamics of other drugs used for treatment of symptomatic HF, specifically between the TNF alpha inhibitors and medications employed for CHF [30]. These drugs include angiotensin-converting enzyme (ACE) inhibitors, beta-blockers, and diuretics [30]. Further studies may be indicated for these interactions.

## Conclusions

TNF alpha inhibitors have been effective in controlling and managing patients with RA, but the risk of elevated cardiovascular risk continues to be debated. We have found that while inhibition of TNFR1, an apoptotic receptor, is cardioprotective, the greater inhibition of TNFR2, which is a cardioprotective receptor, may be the cause of the cardiovascular risks associated with TNF alpha inhibitors. Based on our findings and the role TNFR1 and TNFR2 receptors play, we recommend further study of the biochemical pathways of TNF alpha, which will further allow for the synthesis of selective TNFR1 inhibitors or TNFR2 agonists as treatment modalities for patients with RA and concomitant cardiovascular disease. In the meantime, proper patient risk factor analysis, specific testing protocols, and greater communication between cardiologists and rheumatologists should improve the management of patients with RA and at cardiovascular risk. Treatment decisions should be based on this risk and managed closely to prevent serious medication-induced cardiovascular complications.
